# HPC: Hierarchical phylogeny construction

**DOI:** 10.1371/journal.pone.0221357

**Published:** 2019-08-22

**Authors:** Anindya Das, Xiaoqiu Huang

**Affiliations:** Department of Computer Science, Iowa State University, Ames, Iowa, United States of America; Washington State University, UNITED STATES

## Abstract

Rapid improvements in DNA sequencing technology have resulted in long genome sequences for a large number of similar isolates with a wide range of single nucleotide polymorphism (SNP) rates, where some isolates can have thousands of times lower SNP rates than others. Genome sequences of this kind are a challenge to existing methods for construction of phylogenetic trees. We address the issues by developing a hierarchical approach to phylogeny construction. In this method, the construction is performed at multiple levels, where at each level, groups of isolates with similar levels of similarity are identified and their phylogenetic trees are constructed. Time savings are achieved by using a sufficiently large number of columns from the input alignment, instead of all its columns. Our results show that the new approach is 20-60 times more efficient than existing programs and more accurate in situations where highly similar isolates have a wide range of SNP rates.

## Introduction

Phylogenetic analysis is useful in revealing the evolutionary relationships among isolates (strains) from different species. Most phylogenetic inference methods can be classified into three categories: distance matrix methods [1], parsimony methods [2] and maximum likelihood methods [3]. Maximum likelihood methods use an explicit model of sequence evolution in estimating phylogenetic trees from a multiple sequence alignment for the isolates [4]; parsimony methods operate on an implicit model of evolution [5]. Distance matrix methods take as input a matrix of distances, one for each pair of isolates [6], which can be computed from pairwise sequence alignments, with or without a model of evolution [7], or computed from sequences, without any alignments or model of evolution [8]. Alignment free methods are useful in situations where sequences are too divergent to be accurately aligned [9]. Note that maximum likelihood methods cannot be used in such situations, because of lack of an accurate multiple sequence alignment. On the other hand, when the sequences of isolates are similar enough to be accurately aligned, distance matrix methods are more efficient but less accurate than maximum likelihood methods [10]. Our approach intends to achieve the efficiency of distance matrix methods while retaining the accuracy of maximum likelihood methods.

Due to tremendous advances in next-generation sequencing, genome sequences can be produced for a large number of similar isolates with a wide range of single nucleotide polymorphism (SNP) rates, where some isolates can have thousands of times lower SNP rates than others [11]. Genome alignments in the sizes of millions to billions of base pairs can be produced by mapping short reads to a reference sequence [12, 13]. Such huge genome alignments are a challenge to existing methods for construction of phylogenetic trees, because popular programs, e.g. RAxML [14], IQ-TREE [15], FastTree [16], and PhyML [17], compute the maximum likelihood for every column of the input alignment. We address these two issues by developing a hierarchical approach to phylogeny construction.

In this method, the construction is performed at multiple levels. At the top level, a group of isolates with the lowest levels of similarity are identified and their phylogenetic tree is constructed. At the next level, groups of isolates with much (e.g. 100 times) higher levels of similarity are considered. If there exist groups of isolates with greatly (e.g. 10,000 times) higher levels of similarity, they are considered at the subsequent levels. This hierarchical approach is more likely to produce trees with less variation in branch length. Time savings are achieved by using a sufficiently large number of columns from the input alignment, instead of all its columns. Our results on datasets generated by simulation show that the new approach is 20-60 times more efficient than existing programs and more accurate in situations where highly similar isolates have a wide range of SNP rates.

## Methods

Our method (HPC) takes as input an alignment for a set of isolates, and proceeds in the following steps.

1. Partition the input set of isolates into a group of moderately similar isolates and subgroups of highly similar isolates such that all isolates in every subgroup are highly similar to the same isolate (called a representative isolate) in the group.
2. Form a proper alignment for the group from the input alignment by selecting, for each isolate in the group, its alignment row.
3. Construct a phylogeny for the group of isolates with its alignment.
4. Process the subgroups:
  1. If no subgroup contains any isolates, then terminate.
  2. Otherwise, for each subgroup of isolates, add its representative isolate to the subgroup, form a proper sub-alignment for the subgroup, and recursively apply the above steps to the sub-alignment.

Below we describe the partition and phylogeny construction in detail.

### Partition of isolates

We use an evolutionary distance-based method for identifying moderately similar isolates. The evolutionary distance *D*_*p*,*q*_ between two isolates *p* and *q* can be computed by applying a maximum-likelihood method on their pairwise sequence alignment [7]. Let *D*_*max*_ denote the maximum of the evolutionary distances between all pairs of isolates. Two isolates *p* and *q* are highly similar if the ratio *D*_*max*_/*D*_*p*,*q*_ is greater than a distance ratio cutoff *δ*. In practice, we can use a more efficient method for obtaining a value close to *D*_*max*_. In this method, we randomly select an isolate and compute the evolutionary distance between this isolate and every other isolate. Let ${\hat{D}}_{max}$ denote the maximum of the computed evolutionary distances. It can be easily shown that ${\hat{D}}_{max}\leq D_{max}\leq 2{\hat{D}}_{max}$, so ${\hat{D}}_{max}$ is used in place of *D*_*max*_. Another efficiency improvement is to replace the evolutionary distance between two isolates by the percent difference of their pairwise alignment.

**Fig 1:**
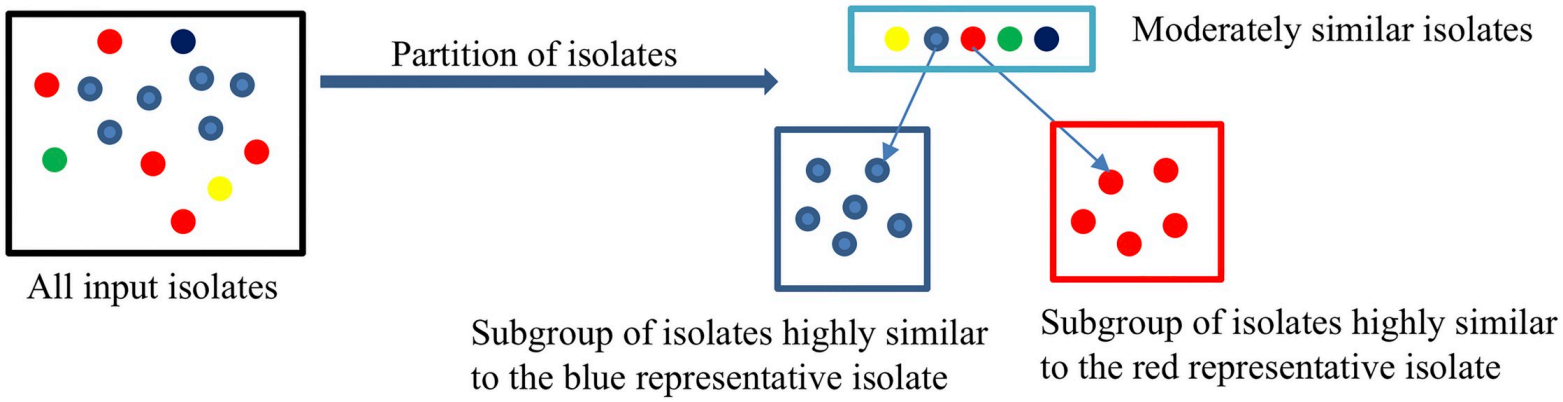
Illustration of the partition step. A collection of isolates (colored ovals) is partitioned into a group of moderately similar isolates and two subgroups of highly similar isolates.

Next we form a group *H* of moderately similar isolates such that no two isolates in the group are highly similar. Initially one isolate is chosen at random as a first element of *H*. Then for every other isolate, if it is not highly similar to any isolate in *H*, then it is added to *H*. Otherwise, it is placed into a subgroup of isolates that were each highly similar to an isolate in *H*. Thus, the partition step produces the group *H* and a subgroup of highly similar isolates for each isolate in *H*. Note that some subgroups may be empty. See Fig 1 for an illustration of this step.

### Construction of phylogeny for the current group

We describe a phylogeny construction step for the current group of isolates with its alignment *S*. Let *d*_*S*_ be the number of columns each with different nucleotides (or letters) in the alignment. If the number of identity columns (without different nucleotides) in the alignment is greater than the maximum number *β* * *d*_*S*_ of allowed identity columns, then the extra number of identity columns are removed from the alignment. Then we form a smaller alignment by randomly selecting *α* columns without replacement from the alignment. Next we construct a phylogeny for the current group of isolates by using an existing method on the smaller alignment. Finally we examine the phylogeny by checking if the ratio of the maximum branch length to every branch length in the phylogeny is below the distance ratio cutoff *δ*. If so, the construction terminates. Otherwise, double the value *α* and repeat the process until it is not possible to reduce the number of branches whose ratios are above the cutoff *δ* or the whole alignment is used in the construction. The values for the parameters *α*, *β* and *δ* are determined based on the number of isolates in the group by simulation.

### Values for parameters of HPC

The value for the distance ratio cutoff *δ* was set to 100, because of the way in which the datasets were generated. Another option was to set the value to 50 to deal with a situation in which ${\hat{D}}_{max}$ was close to *D*_*max*_/2 so that estimated distance ratios were smaller than actual ones by a factor of 2. The value for the parameter *β* was simply set to ∞ for the group of moderately similar isolates and 0 for each subgroup of highly similar isolates. Of course, more appropriate values for *β* could be selected by simulation (see the following example).

We used simulation to select a proper initial value for the parameter *α*, which is the number of alignment columns used to construct a phylogeny. True phylogenies for various numbers of isolates and alignments of various lengths were generated. Then the whole alignments were used to build phylogenies with existing programs, and the Robinson-Foulds distances [18] between the true and constructed phylogenies were computed to assess the effect of the alignment length and the number of isolates on the Robinson-Foulds distance. Given the number of isolates, we intended to select the number of alignment columns (the value for the parameter *α*) that corresponds to a topology with a small Robinson-Foulds distance. Let *n* be the number of isolates. The initial value for *α* was set to 5, 000 for *n* ≤ 40, to 15, 000 for 40 < *n* ≤ 60, and to 25, 000 otherwise.

### Values for parameters of the existing programs

The three existing programs RAxML, IQ-TREE and FastTree were used alone on the generated datasets with the same set of parameter values as they were called as part of our HPC program. The general reversible process (GTR) model was used as the model of nucleotide substitution, along with a request of 16 threads, for the three programs. For RAxML, we selected the “-f a” option to run a full analysis, 40 as the number of alternative runs on distinct starting trees, 44701 and 65701 as the random number seeds for parsimony inference and bootstrapping, respectively. The default values were used for the other parameters of the three programs.

### Computational resources and average wall-clock time

We used a Linux cluster with a large number of compute nodes at Iowa State University to run IQ-TREE and *HPC*_*IQ*_ on all datasets. A computation job by each program on every dataset was submitted to one of the compute nodes in the cluster, where each node consists of two 8-core, 2.6 GHz Intel Haswell E5-2640 v3 processors (a total of 16 cores). The average wall-clock time taken by all the jobs by the same program on the datasets of the same type was obtained.

We used a different Linux server to process computation jobs by each of the programs *HPC*_*RAx*_, FastTree and *HPC*_*Fast*_ on every dataset of each type. The jobs were submitted to the server one at a time, requesting 16 threads, where the server has 24 cores, 2.90 GHz Intel Xeon E5-4617 processors. The average time of all the jobs by the same program on the datasets of the same type was calculated.

## Results

We evaluated the HPC and three existing programs on datasets generated by simulation over given phylogenies (as the correct answers). We generated 200 datasets each with 350 isolates of *Narrow* type, and 300 datasets each with 590 isolates of *Wide* type, where a major difference between the two dataset types was that the branch lengths in the parts of the phylogeny for highly similar isolates were larger and had wider variations for the *Wide* type than for the *Narrow* type. In each type of datasets, the alignment length was one million columns, and there were a group of 50 moderately similar isolates and 24 subgroups of highly similar isolates. However, the size of each subgroup was 10 or 15 for the *Narrow* type, and 10, 15, 20, 25, 30 or 35 for the *Wide* type. We have used three existing programs: RAxML, IQ-TREE and FastTree and our hierarchical approach on these generated datasets.

### Generation of datasets

A simulated dataset was generated as follows. A true phylogeny for a group of 50 moderately similar isolates was randomly created, and an alignment with one million columns was generated with the Seq-Gen program [19] by simulating the evolution of nucleotide sequences over the phylogeny according to the general reversible process (GTR) model of substitution [20]. We selected 0.05, 0.10, 0.15, 0.20, …, 0.95 mutations per site (with equal probability) as branch lengths in the true phylogeny for the group of moderately similar isolates in both types of datasets. Each isolate in the group of moderately similar isolates was made available as a potential representative for a subgroup of highly similar isolates. Then for each of the 24 subgroups of highly similar isolates, a true phylogeny was randomly created, an alignment with one million columns was generated, a representative was randomly selected from the moderately similar isolates and made unavailable for subsequent selections, and the alignment was adjusted at each identity column by replacing each letter in the column with the letter in the corresponding column of the representative isolate. The distribution of branch lengths used for moderately similar isolates was also used for generating the alignment for highly similar isolates in *Wide* datasets. Initially, alignments with 2500, 3500, 5000, 6500, 8000 and 9500 columns were created according to the true phylogenies consisting of 10, 15, 20, 25, 30 and 35 highly similar isolates, respectively. Then each of these alignments was extended to one million columns by adding identity columns. For each *Narrow* dataset, we selected 0.0001, 0.00009, 0.00007, 0.00005, 0.00003, 0.00001, 0.000005, 0.000003, 0.000001 mutations per site (with equal probability) as branch lengths in the true phylogeny for each subgroup of 10 or 15 highly similar isolates to generate an alignment with one million columns. For both *Wide* and *Narrow* datasets, any group of highly similar isolates had sufficient number of identical columns among themselves so that the percent identity of any two highly similar isolates was at least 100 times higher than that of any two moderately similar isolates.

### Comparison of HPC with existing programs

Each of the three existing programs RAxML, IQ-TREE and FastTree was adapted as a phylogeny construction engine inside HPC, resulting in three HPC versions: *HPC*_*RAx*_, *HPC*_*IQ*_ and *HPC*_*Fast*_. Each HPC version along with each existing program was evaluated on each of the generated datasets in efficiency and accuracy. Shown in Table 1 is the average time required by each of these programs on each type of generated datasets.

**Table 1:** Time required by three versions of HPC and three existing programs.

Data	RAxML	*HPC*_*RAx*_	IQ-TREE	*HPC*_*IQ*_	FastTree	*HPC*_*Fast*_
Narrow	> 168 hr	12 hr	31 hr	35 min	5 hr	15 min
Wide	> 168 hr	15 hr	60 hr	1 hr	8 hr	20 min

Next, the accuracy of each program was assessed by computing the normalized Robinson-Foulds distance [18] between the true and constructed phylogenies for each subgroup of highly similar isolates and calculating the sum of these distances over all 24 subgroups. The Robinson-Foulds distance between two unrooted trees (each with *n* leaves) is the number of partitions (internal branches) present in one tree but absent in the other. To account for variable tree sizes, the distance is normalized by dividing it by the total number (2*n* − 6) of internal branches in both trees.

**Fig 2:**
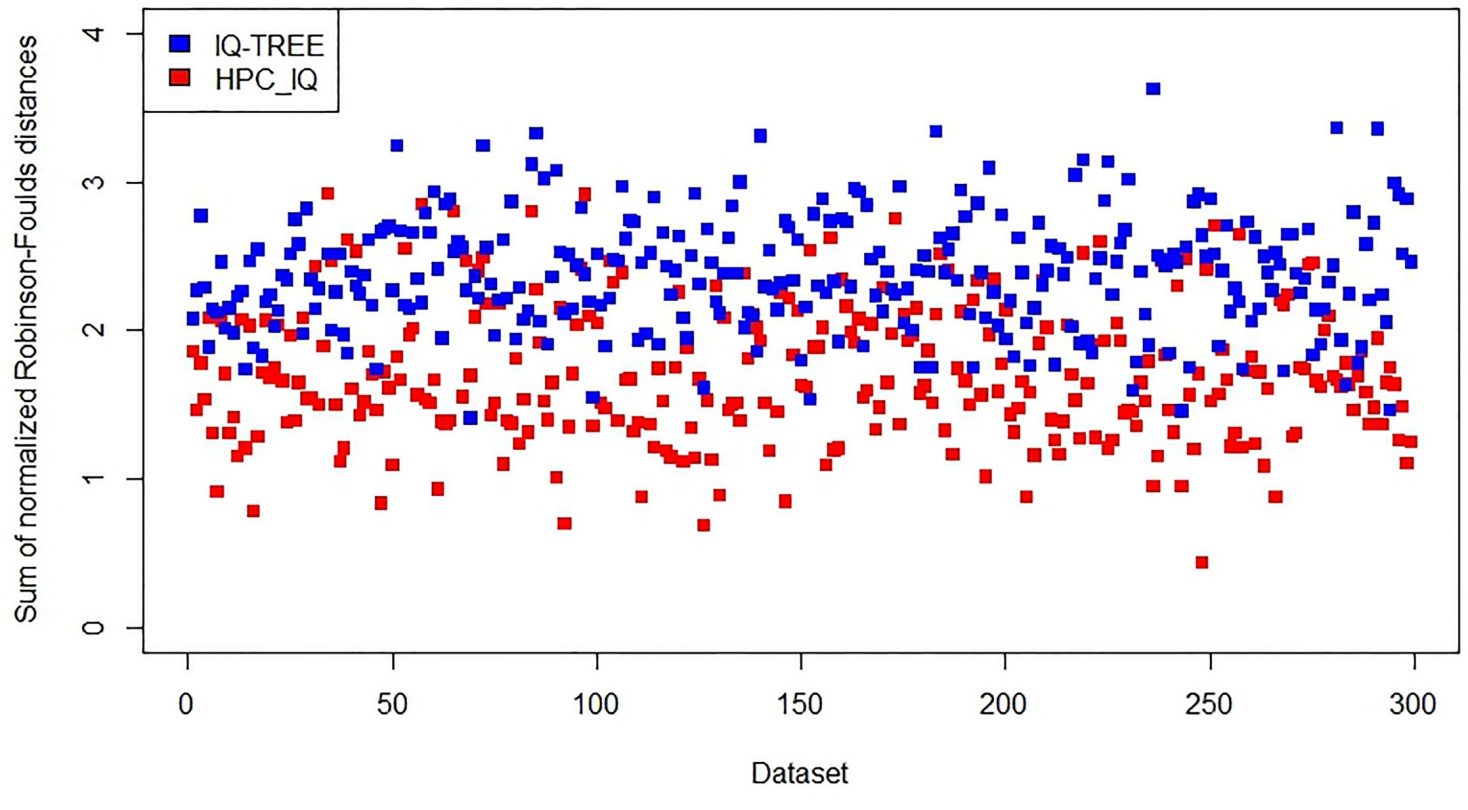
Accuracy assessment of IQ-TREE and *HPC*_*IQ*_ on 300 *Wide* datasets.

**Fig 3:**
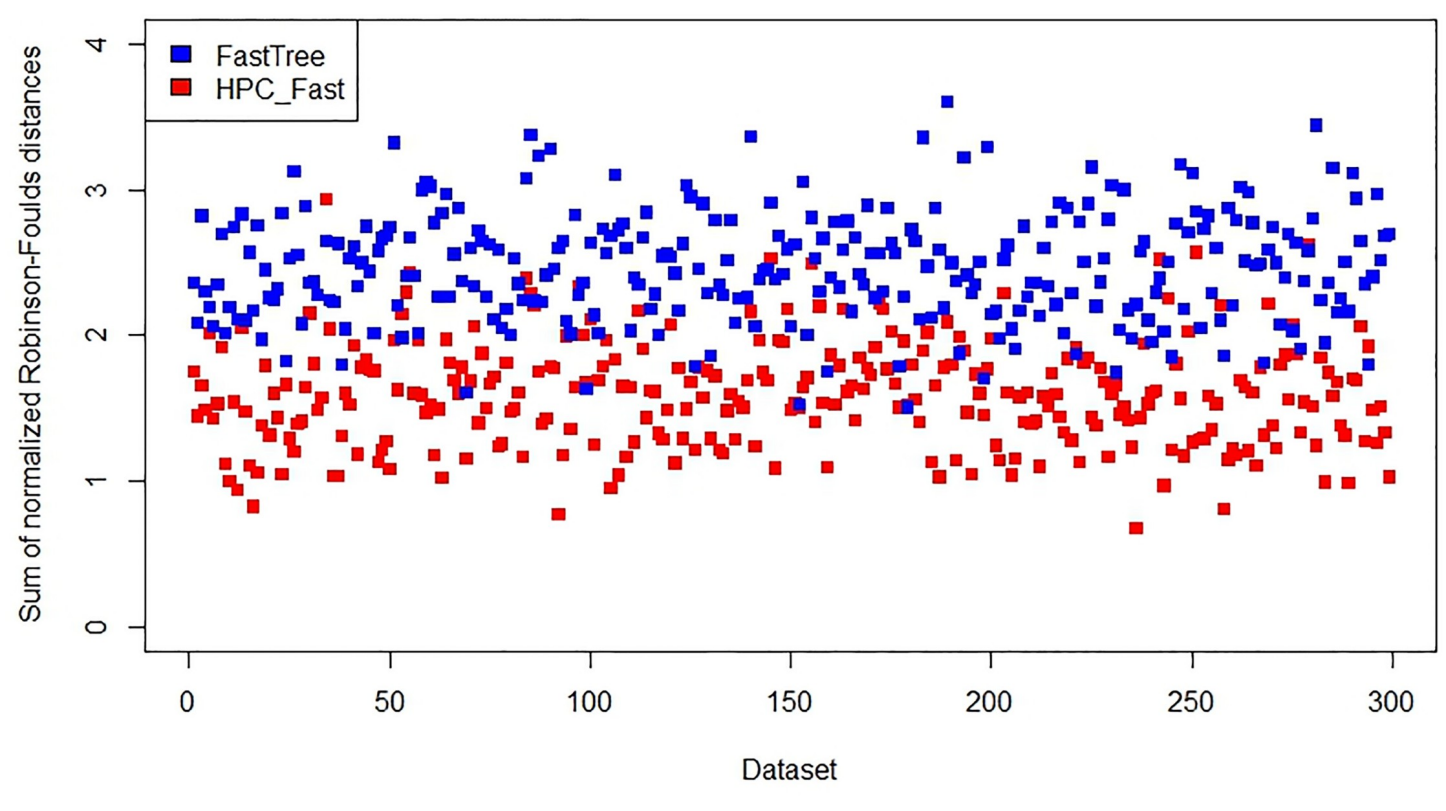
Accuracy assessment of FastTree and *HPC*_*Fast*_ on 300 *Wide* datasets.

The following figures show the results from the existing programs IQ-TREE and FastTree, and from the new programs *HPC*_*IQ*_ and *HPC*_*Fast*_, on the *Wide* and *Narrow* datasets. Because RAxML did not run to completion in the allocated time of 1 week on any of the *Wide* and *Narrow* datasets, we were not able to compare RAxML with *HPC*_*RAx*_ in accuracy. On the *Wide* datasets, the new programs produced more accurate results than the existing programs (Figs 2 and 3).

**Fig 4:**
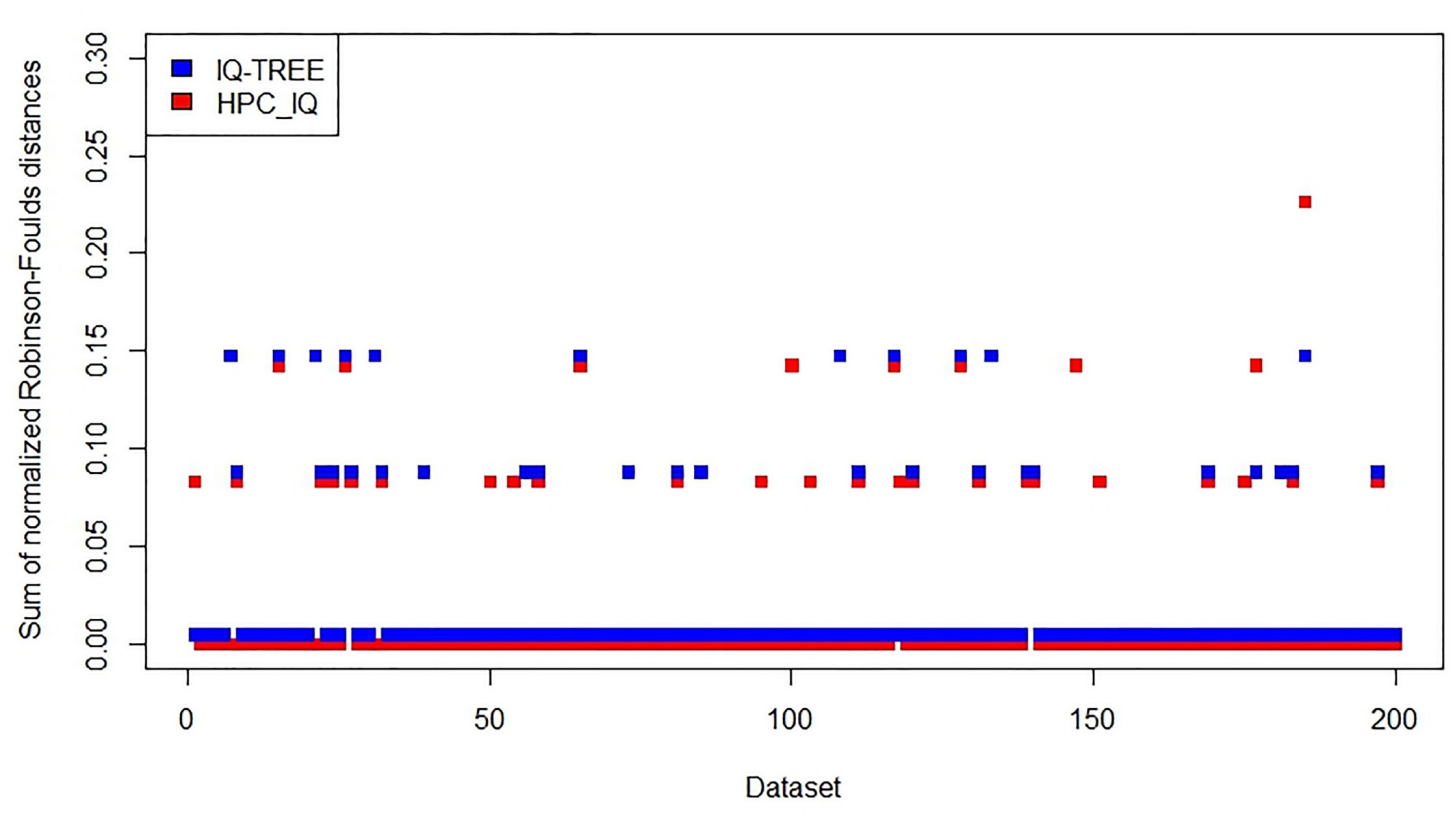
Accuracy assessment of IQ-TREE and *HPC*_*IQ*_ on 200 *Narrow* datasets.

**Fig 5:**
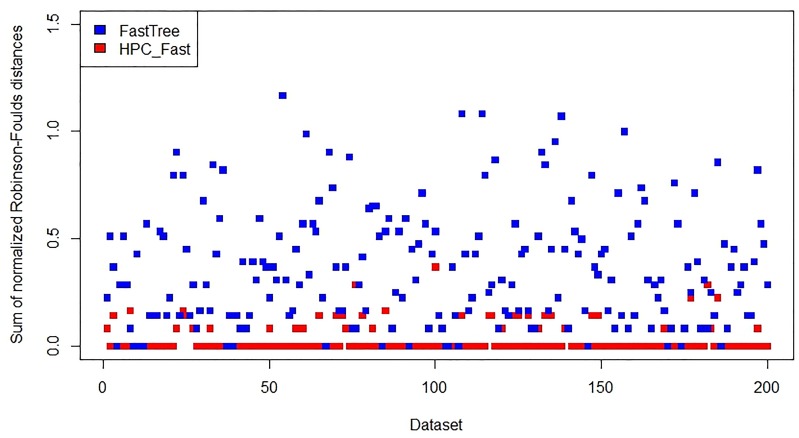
Accuracy assessment of FastTree and *HPC*_*Fast*_ on 200 *Narrow* datasets.

On the *Narrow* datasets, the new program *HPC*_*IQ*_ is similar to the existing program IQ-TREE in accuracy (Fig 4), while the new program *HPC*_*Fast*_ is more accurate than the existing program FastTree (Fig 5). Thus, the results indicate that accuracy has not been lost with the new approach.

A summary of the results is shown in Table 2. We observed no significant differences in accuracy between these programs on the group of moderately similar isolates.

**Table 2:** Average normalized Robinson-Foulds distance for each program.

Data	RAxML^1^	*HPC*_*RAx*_	IQ-TREE	*HPC*_*IQ*_	FastTree	*HPC*_*Fast*_
Narrow	N/A	0.0151	0.0167	0.0164	0.3807	0.0314
Wide	N/A	1.5137	2.3819	1.7151	2.4674	1.5938

^1^ RAxML did not run to completion in the allocated amount of time.

We randomly selected a *Wide* dataset with 590 isolates to illustrate trees produced by *HPC*_*Fast*_, IQ-TREE and FastTree. On this dataset, *HPC*_*Fast*_ produced a tree at the top level for a group of 50 moderately similar isolates and trees at the next level for each of 24 subgroups of highly similar isolates. The tree at the top level is shown in S1 Fig, and trees at the next level for two subgroups of highly similar isolates are shown in S2 and S3 Figs. Also shown are trees by IQ-TREE and FastTree on the same dataset (S4 and S5 Figs). Note that support values at internodes were computed by FastTree (alone and inside *HPC*) from 1, 000 bootstrap replicates. Both IQ-TREE and FastTree build a flat tree containing all isolates. When the number of isolates is over 500 and the branch lengths of the tree vary over 100-fold, the tree may be difficult to visualize in detail, as shown by this example. *HPC*, on the other hand, constructs a number of smaller trees at multiple levels, instead of a large flat tree. Such trees are easy to visualize in detail, as shown by the example.

### Availability

HPC was implemented in C++. Full implementation with examples and instructions for running the program is available at https://github.com/anindya028/HPC. Also available at https://github.com/anindya028/HPC/tree/master/supporting_information are the one-level trees produced by each of IQ-TREE and FastTree alone on the *Wide* and *Narrow* datasets, the two-level trees produced by each of these programs inside *HPC* on the same datasets, and the true trees used to generate the input datasets. The collections of triplets of numbers used to build Figs 2–5 are given, respectively, in S1–S4 Files in comma-separated values (CSV) format.

## Discussion

It is difficult to construct an accurate phylogenetic tree with millions of isolates. If isolates have a wide range of evolutionary distances such that they can be partitioned into a multiple-level structure with a much smaller number of similarly distanced isolates at every level, our hierarchical approach provides an efficient way to construct a much smaller tree at every level. Clearly, it is less difficult to construct a small tree than a large tree. Another benefit of our approach is that the constructed structure of trees makes it easier for biologists to understand the evolutionary relationship among the isolates than a much larger flat tree does. Although the hierarchical approach is promising, it needs many more improvements to meet the needs of evolutionary biologists.

## Supporting information

S1 Fig Tree at the top level produced by *HPC*_*Fast*_ on a group of 50 moderately similar isolates. The names for all isolates representing nonempty subgroups begin with ‘Group’, while the names for the other isolates begin with ‘Taxon’.
(TIF)

S2 Fig Tree at the second level produced by *HPC*_*Fast*_ on a group (represented by Group3) of highly similar isolates. (TIF)

S3 Fig Tree at the second level produced by *HPC*_*Fast*_ on a group (represented by Group10) of highly similar isolates. (TIF)

S4 Fig Tree produced by IQ-TREE on the same *Wide* dataset with 590 isolates. Note that fewer than 100 isolates are shown on the tree because of its size and structure.
(TIF)

S5 Fig Tree produced by FastTree on the same *Wide* dataset. Note that fewer than 100 isolates are shown on the tree because of its size and structure.
(TIF)

S1 File Collection of triplets of numbers used to build Fig 2. (CSV)

S2 File Collection of triplets of numbers used to build Fig 3. (CSV)

S3 File Collection of triplets of numbers used to build Fig 4. (CSV)

S4 File Collection of triplets of numbers used to build Fig 5. (CSV)
