# Supplementary figures and images for: HPC: Hierarchical phylogeny construction
Source: PLoS One. 2019 Aug 22;14(8):e0221357. doi: 10.1371/journal.pone.0221357 (PMC6705828; doi:10.1371/journal.pone.0221357)

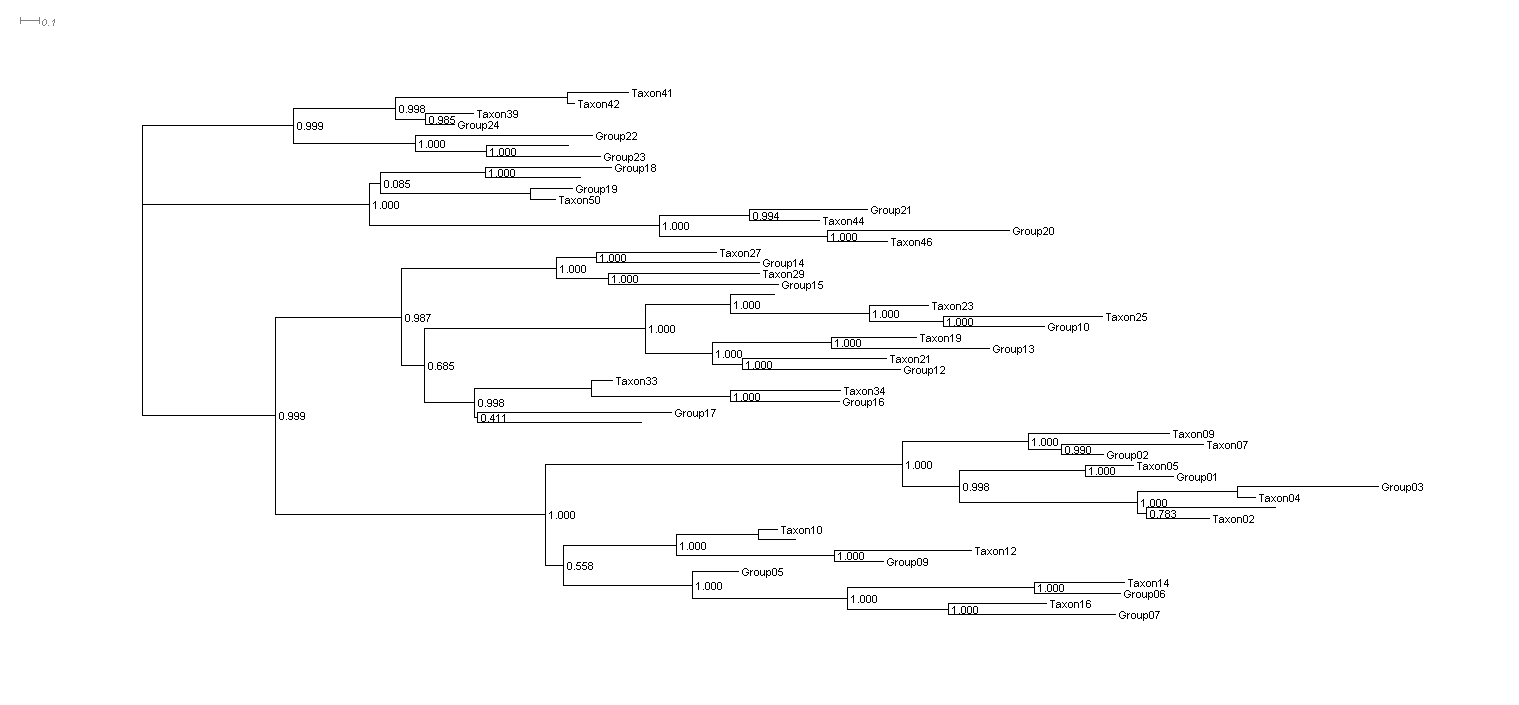

Supplement: S1 Fig — The names for all isolates representing nonempty subgroups begin with ‘Group’, while the names for the other isolates begin with ‘Taxon’. (TIF) [file pone.0221357.s001.tif]

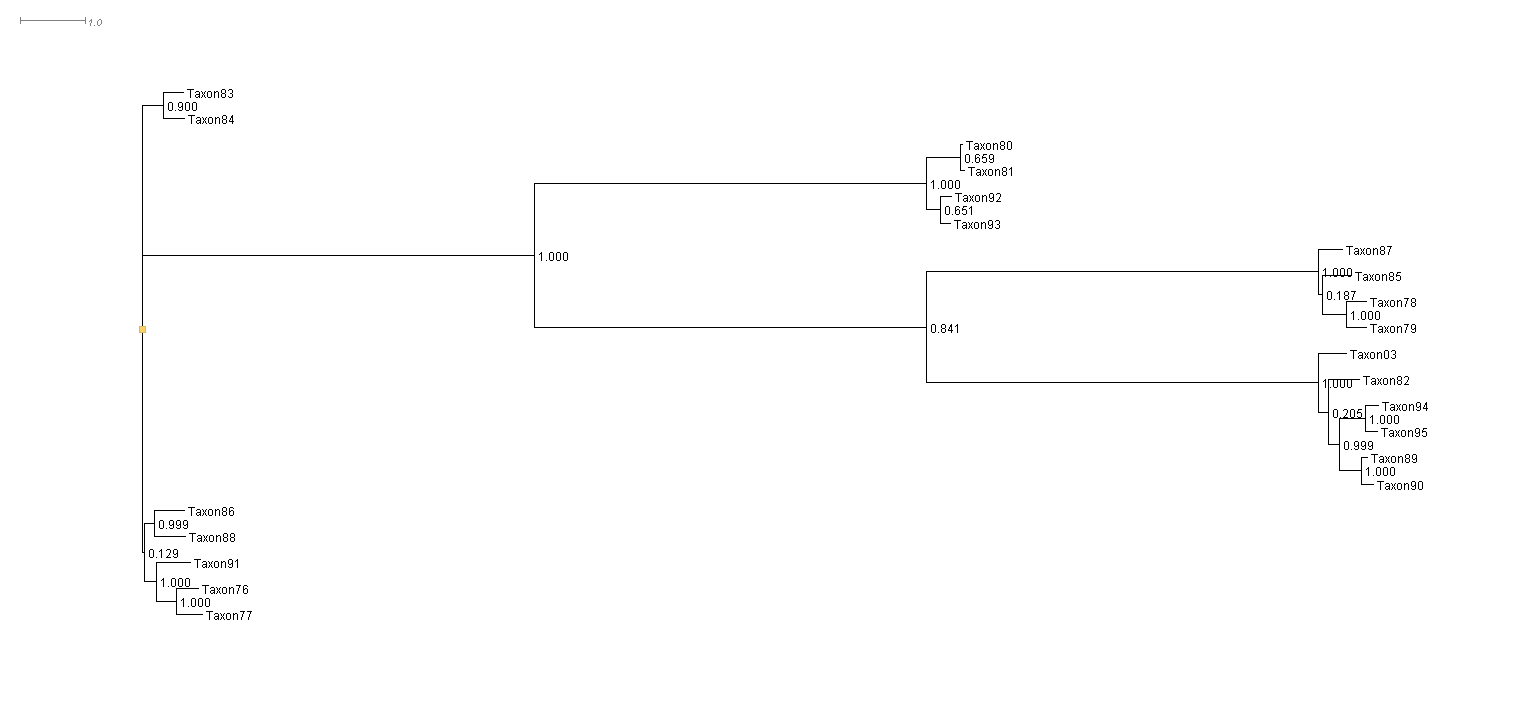

Supplement: S2 Fig — (TIF) [file pone.0221357.s002.tif]

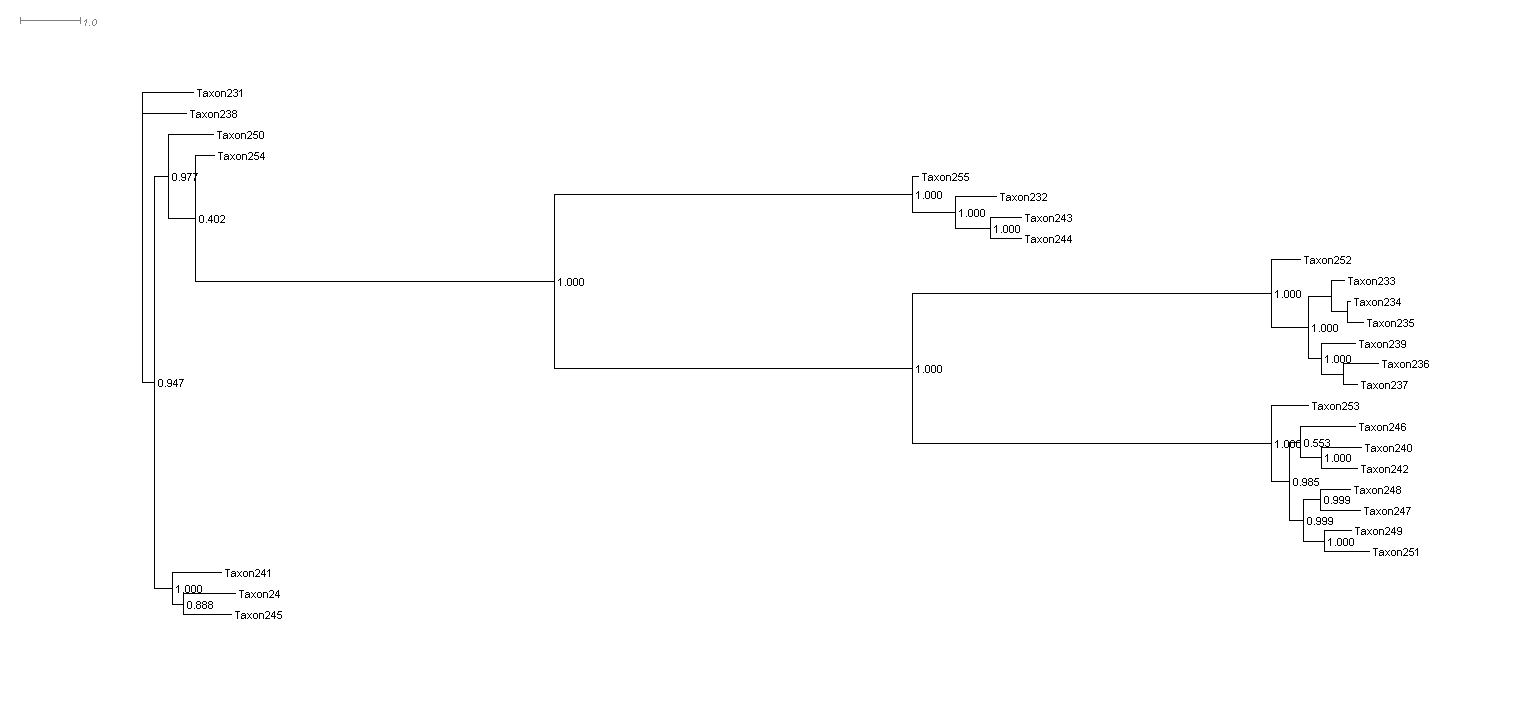

Supplement: S3 Fig — (TIF) [file pone.0221357.s003.tif]

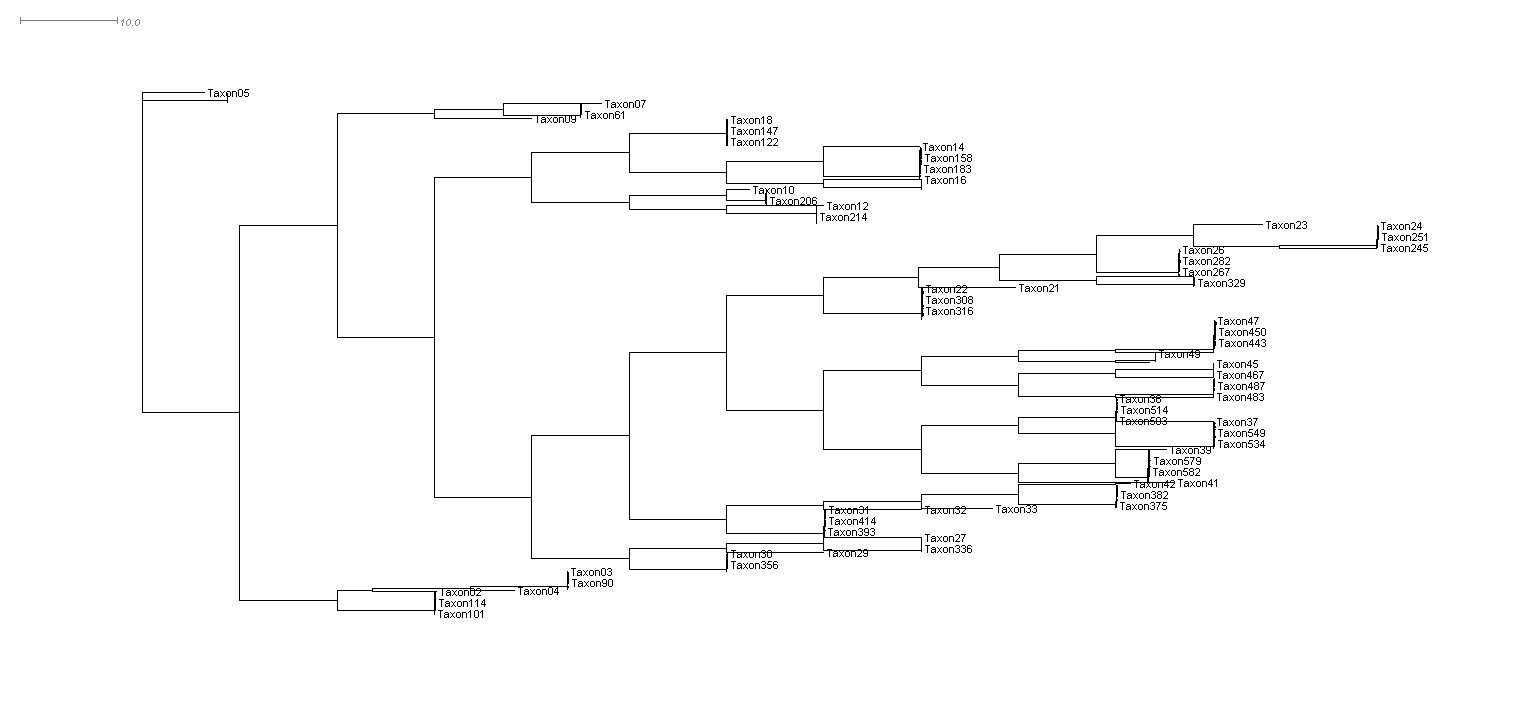

Supplement: S4 Fig — Note that fewer than 100 isolates are shown on the tree because of its size and structure. (TIF) [file pone.0221357.s004.tif]

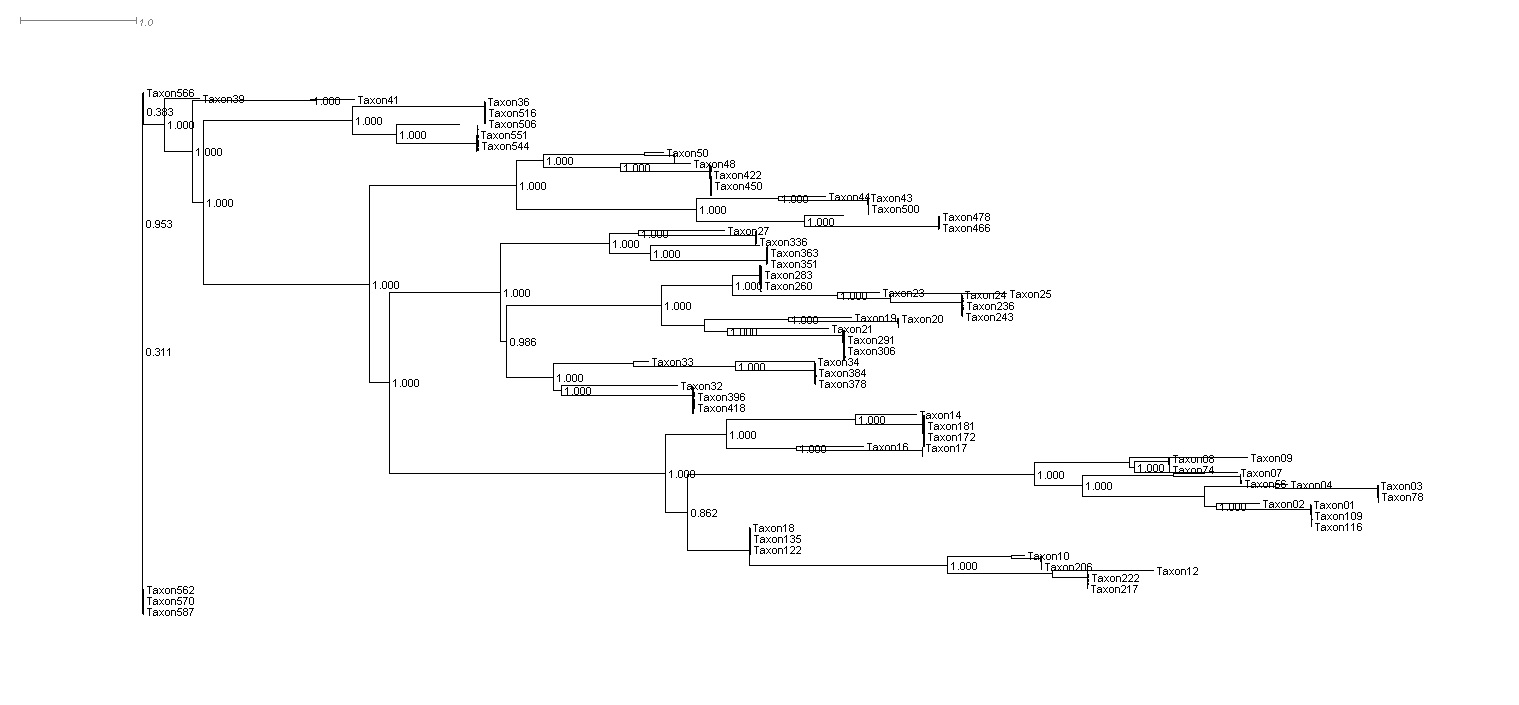

Supplement: S5 Fig — Note that fewer than 100 isolates are shown on the tree because of its size and structure. (TIF) [file pone.0221357.s005.tif]
